# Filling the GAP (DH) in Pre-Clinical Models

**DOI:** 10.1097/HS9.0000000000000311

**Published:** 2019-10-22

**Authors:** Melania Tesio

**Affiliations:** Laboratory of Onco-Hematology, Institut Necker Enfants Malades (INEM), Institut National de Recherche Médicale (INSERM) U1151, Paris, France.

Murine models are instrumental to elucidate the molecular mechanisms that regulate normal and malignant hematopoiesis. Multiple criteria, however, need to be fulfilled for a mouse model to be ideal. To faithfully mimic a hematological malignancy, for instance, a pre-clinical model should develop cancers with high reproducibility and penetrance, replicate the genetic and molecular heterogeneity of the tumor and mimic the clinical behavior of the human disease. Meeting these pre-requisites has been challenging when modeling angioimmunoblastic T-cell lymphoma (AITL), one of the most common form of peripheral T-cell lymphomas, which is highly resistant to conventional chemotherapy and associated to a poor prognosis.

**Figure 1:**
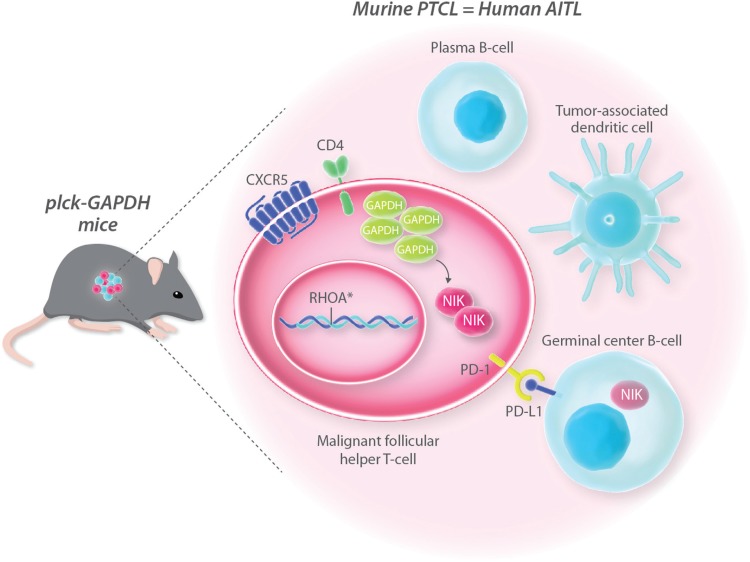
Transgenic mice overexpressing GADPH in T-cells develop a peripheral T-cell lymphoma (PTCL), which recapitulates pathological, immuno-phenotypic, genetic and transcriptional features of human AITL (Figure adapted from Mondragòn et al *Cancer Cell* 2019).

In an elegant study, recently published in *Cancer Cell*, Els Verhoeyen's group overcame the pitfalls limiting previous models of this malignancy^1,2^ and created a model that fully recapitulates the human disease.^3^ The researchers generated a transgenic mouse whereby the glyceraldehyde-3-phosphate dehydrogenase (GAPDH) is over-expressed in T-cells under the control of a T-cell specific promoter (plck). Upon aging, these transgenic mice developed peripheral T-cell lymphomas that recapitulate pathological, immuno-phenotypic, genetic and transcriptional features of human AITL (Fig. 1). Consistent with the clinical manifestation of the human disease, aged GAPDH transgenic mice developed lymphoadenopathy, splenomegaly and hepatomegaly. Moreover, they showed enlarged mesenchymal lymph nodes and manifested autoimmunity-like symptoms. The immunophenotypic characterization of the splenic and lymph node tumors revealed that the neoplastic T-cells have a follicular helper T-cell (TFH) phenotype (CD4^+^PD-1^high^CXCR5^+^), which is one of the hallmarks of human AITL. These malignant T-cells associated to germinal center B-cells (GC), plasma B-cells and tumor-associated follicular dendritic cells, thus reproducing the peculiar tumor microenvironment observed in human AITL (Fig. 1). Along this line, murine lymphomas showed TFH cells, CG B-cells and plasma B-cells gene signatures and up-regulated inflammation and humoral immune response genes, thus presenting an expression profile closely matching the transcriptional profile observed in a cohort of 60 AITL patients. Last, but not least, murine lymphomas also reproduced main genetic lesions observed in AITL patients, such as a mutation in the small guanosine triphospate GTPase RHOA, which undergoes loss of function mutations in 50% to 70% of AITL patients.

By faithfully recapitulating major features of the human disease, the GAPDH overexpressing model developed by Mondragòn et al constitutes an excellent model for drug testing and development. This is foremost important in AITL, whose poor prognosis (30% survival rate at 5 years) calls for novel therapeutic treatment. Mimicking the strong activation of the NF-kB pathway observed in primary AITL cells, murine tumors strongly up-regulated the NIK kinase, which is key to activate the non-canonical NF-kB signaling. These findings promoted the authors to develop a substituted halo-quinoline derivative, which potently inhibited NIK activity. This novel drug significantly increased the survival of immune-deficient mice engrafted with GADPH-overexpressing tumors. In addition, it strongly reduced the growth of CD4^+^ TFH cells and GC B-cells isolated from human AITL biopsies.

These data are intriguing as they provide a rationale for the use of NF-KB inhibitors in AITL. As evidenced ex vivo, moreover, NF-kB inhibition may provide significant therapeutic effects by targeting not only tumor cells but also components of the microenvironment, such as GC B-cells. This is an interesting aspect that warrants further investigations, especially in the light of the prognostic impact recently linked to microenvironment-related signatures in AITL.^4^ Given NF-kB essential role in drug-resistance in other cancers as well as the strong chemotherapy resistance observed in AITL patients, it will be also interesting to determine whether the novel drug developed by Mondragòn et al can, at least in part, sensitize AITL cells to chemotherapeutic agents. Last, the work by Mondragòn et al offers a powerful model to unequivocally verify whether follicular helper T-cells are indeed cells of origin in AITL, an aspect so far mostly interfered by gene expression profile studies. GAPDH transgenic mice will thus certainly provide novel important insights into the biology of AITL and its therapeutic options.
